# Multiple Light-Activated Photodynamic Therapy of Tetraphenylethylene Derivative with AIE Characteristics for Hepatocellular Carcinoma via Dual-Organelles Targeting

**DOI:** 10.3390/pharmaceutics14020459

**Published:** 2022-02-21

**Authors:** Chuxing Chai, Tao Zhou, Jianfang Zhu, Yong Tang, Jun Xiong, Xiaobo Min, Qi Qin, Min Li, Na Zhao, Chidan Wan

**Affiliations:** 1 Department of Hepatobiliary Surgery, Union Hospital, Tongji Medical College, Huazhong University of Science and Technology, Wuhan 430022, China; chaichuxing@hotmail.com (C.C.); tangyong_2222@126.com (Y.T.); oldxiong@163.com (J.X.); d201981629@hust.edu.cn (X.M.); qinqi8441@163.com (Q.Q.); 2 Department of Otorhinolaryngology, Union Hospital, Tongji Medical College, Huazhong University of Science and Technology, Wuhan 430022, China; entzt@hust.edu.cn; 3 Central Laboratory, Union Hospital, Tongji Medical College, Huazhong University of Science and Technology, Wuhan 430022, China; 342244204@aliyun.com; 4 Key Laboratory of Macromolecular Science of Shaanxi Province, School of Chemistry & Chemical Engineering, Shaanxi Normal University, Xi’an 710119, China; nzhao@snnu.edu.cn; 5 Key Laboratory of Applied Surface and Colloid Chemistry of Ministry of Education, School of Chemistry & Chemical Engineering, Shaanxi Normal University, Xi’an 710119, China; 6 Key Laboratory of the Ministry of Education for Medicinal Resources and Natural Pharmaceutical Chemistry, Shaanxi Normal University, Xi’an 710119, China

**Keywords:** photodynamic therapy, aggregation-induced emission, organelles targeting, hepatocellular carcinoma

## Abstract

Photodynamic therapy (PDT) has emerged as a promising locoregional therapy of hepatocellular carcinoma (HCC). The utilization of luminogens with aggregation-induced emission (AIE) characteristics provides a new opportunity to design functional photosensitizers (PS). PSs targeting the critical organelles that are susceptible to reactive oxygen species damage is a promising strategy to enhance the effectiveness of PDT. In this paper, a new PS, 1-[2-hydroxyethyl]-4-[4-(1,2,2-triphenylvinyl)styryl]pyridinium bromide (TPE-Py-OH) of tetraphenylethylene derivative with AIE feature was designed and synthesized for PDT. The TPE-Py-OH can not only simultaneously target lipid droplets and mitochondria, but also stay in cells for a long period (more than 7 days). Taking advantage of the long retention ability of TPE-Py-OH in tumor, the PDT effect of TPE-Py-OH can be activated through multiple irradiations after one injection, which provides a specific multiple light-activated PDT effect. We believe that this AIE-active PS will be promising for the tracking and photodynamic ablation of HCC with sustained effectiveness.

## 1. Introduction

Locoregional ablation therapy was recommended as an effective treatment for patients with primary hepatocellular carcinoma (HCC) and liver metastases [1,2]. The mechanism of ablation is to cause the destruction of local tumor by physical or chemical damage. Thermal ablation, including radiofrequency ablation and microwave ablation, has been applied as a safe, low-cost, effective alternative of surgery in small and multifocal liver tumor [3]. The ablation procedure often relies on the placement of the needle electrode into target tumor under the guidance of imaging technology which requires additional competence and fails to provide real-time self-monitoring. Multiple treatment sessions and repetitive puncture operation may be required due to difficult tumor conditions and limitation of imaging technology, which may cause additional suffering of patients [4]. Alternatively, photodynamic therapy (PDT) is a potential tumor-ablative locoregional therapy [5,6,7]. Fluorescent molecules with photodynamic effect can provide precise and safe ablation of tumor at the cellular level while enabling dynamic monitoring of tumor lesion, which has great promise in building cancer-targeted theragnostic platforms [8,9]. PDT can induce both cancer cell death and immune response against the tumor [10,11]. Additionally, intraoperative fluorescence image-guided surgery combined with PDT can eliminate residual tumors and reduce the recurrence of cancer [12,13,14,15]. The use of PDT has gained much attention in the treatment of HCC, and has been approved as a feasible approach for HCC treatment in preclinical studies [6,16].

The three crucial elements of PDT are photosensitizer (PS), light irradiation, and oxygen. PS is the core element of PDT. The procedure of PDT involves administration of PSs which accumulate in the tumor tissue, followed by local illumination with specific wavelength to active PSs [17]. After exposure to the light, the PSs can transfer energy from light to molecular oxygen, contribute to their change of energy states, to generate reactive oxygen species (ROS), which then induce apoptosis, necrosis, and autophagy in treated cells and cause cell death [18,19]. However, traditional PSs—such as porphyrin, phthalocyanine, and analogs—are prone to aggregate in the treated cells or aqueous solution, which will lead to a dramatic reduction in ROS production and suppression in fluorescence emission [20,21].

In 2001, Tang’s group discovered a novel fluorogen with the opposite characteristics, namely aggregation-induced emission (AIE) [22], which is nonemissive in a molecularly dissolved state, but is highly emissive in their aggregate/solid state due to the mechanism of restriction of intramolecular motions/rotations [23]. Meanwhile, as organic molecules, AIE luminogens (AIEgens) were highly editable. The different additional groups and molecular structures can provide different optical, physical, and chemical properties to AIEgens, which enables broad biological medicine application [24,25,26,27]. A series of AIEgens have been developed for translational applications in sensing, imaging, and theranostics with excellent performance than conventional fluorescent probes [28,29]. Recently, some AIEgens were designed to exhibit excellent photosensitization and ROS generation ability, which broadens their potential applications in PDT of cancer. The unique features of AIEgens provide new opportunities for the facile design of PS with special function, high accuracy, and efficacy for image-guided PDT [30,31,32,33].

A critical limitation of PDT is that the half-life of ROS (<40 ns) is usually very short so that it can only act on the production site (<20 nm) [34], which means simple transportation PSs into tumor tissues or cancer cells are not efficient enough to achieve expected outcomes [34,35]. Designing PSs to target the critical organelles that are susceptible to ROS damage is a promising strategy to enhance the effectiveness of PDT. The mitochondrion is a promising target for PSs owing to its crucial role in ROS generation, oxidation-reduction status modulation, and cell apoptosis mediation. Mitochondria in tumor cells are sensitive to ROS generation induced by PDT, which ultimately induces tumor cell apoptosis by disrupting the balance of mitochondrial ROS [36]. Recently, several AIEgens have been designed for targeting mitochondria and have exhibited remarkable photostability, high brightness, and excellent PDT effect [37,38,39,40]. However, multiple doses and repeated injections of PSs are always required for better outcomes in PDT procedures, which may lead to superfluous side effects and additional toxicity to patients [36]. The destruction of tumor vessels of PDT may also reduce the accumulation of PSs in tumor tissue and influence the efficiency of second PDT. Lipid droplets (LDs), also known as adiposomes or lipid bodies, are intracellular lipid-rich organelles for the long-term storage of lipids [41]. The synthesis of lipid droplets is at a lower level in normal cells, whereas tumor cells actively synthesize lipid droplets owing to their high-metabolism state [42,43]. Due to the suitable metabolic activity and stability, as well as their interactions with other organelles, especially mitochondrial, LDs can be valuable targets for PDT [44,45]. Therefore, AIEgens that could dual-target mitochondria and LDs may provide long-term photodynamic effect and better outcomes in PDT treatment procedure after administration of single-dose PSs.

In this work, for developing a new PS with multiple light-activated photodynamic effect and organelle-targeting abilities, the 1-[2-hydroxyethyl]-4-[4-(1,2,2-triphenylvinyl)styryl]pyridinium bromide (TPE-Py-OH) was designed and synthesized. TPE-Py-OH possesses a typical AIE character and exhibits strong fluorescence emission in the aggregated state. It could simultaneously target LDs and mitochondria in living cells. Due to its lipophilic feature, it can aggregate and store in LDs in a relatively stable state for long-term intracellular retention. Meanwhile, TPE-Py-OH also has efficient ROS generation ability and PDT effect both in vitro and in vivo. All these features suggest its superior performance in long-term tracking of living cancer cells and organelles-targeted PDT effect of cancer cells. Benefiting from the strong fluorescence, good photostability, and efficient ROS generation ability of TPE-Py-OH, the long-term cancer cell tracking and multiple light-activated PDT effect of HCC for more than 7 days were achieved after one PS administration. This AIE-active PS exhibit sustained effectiveness in vitro and in vivo, which suggests that the PS can hopefully be applied in long-term intracellular organelle imaging and multiple light-activated photodynamic ablation of HCC.

## 2. Materials and Methods

### 2.1. Materials and Instruments

4-Methylpyridine, 2-bromoethanol, piperidine, anhydrous acetonitrile, and ethanol were purchased from Sigma-Aldrich and used without further purification unless specified otherwise. 4-(1,2,2-Triphenylvinyl)benzaldehyde was synthesized according to the literature method [46,47]. Other chemicals and solvents were all purchased from Aldrich and used as received without further purification. Distilled water was used throughout the experiments. HepG2 cells were obtained from Shanghai Cell Bank of Chinese Academy of Sciences (Shanghai, China). Dulbecco’s modified Eagle medium, fetal bovine serum (FBS), BODIPYTM 493/503 Dye, and JC-1 Dye were purchased from Thermo Fisher Scientific (Waltham, MA, USA). Cell Counting Kit-8 (CCK8) was purchased from Dojindo Molecular Technologies, Inc. (Mashikimachi, Japan). Reactive oxygen species (ROS) assay kit, calcein AM/PI double stain kit, Annexin V-FITC/PI double stain kit and MitoTracker Red FM was purchased from Beyotime Biotechnology (Shanghai, China). Annexin V-FITC/PI apoptosis detection kit was purchased from Qcbio Science & Technologies (Shanghai, China). Tissue culture flask, 96-well plates, and 24-well plates were purchased from Corning Incorporated (Corning, NY, USA). Confocal dishes were purchased from Biosharp Life Science (Hefei, China).

^1^H and ^13^C NMR spectra were measured on a Bruker ARX 400 NMR spectrometer (Bruker, Tucson, AZ, USA) using DMSO-*d_6_* as solvents. High-resolution mass spectra (HRMS) were recorded on a Finnigan MAT TSQ 7000 Mass Spectrometer System (Finnigan MAT, San Jose, CA, USA) operating in a MALDI-TOF mode. UV–vis absorption spectra were recorded on a Biochrom UV visible spectrometer (Biochrom, Berlin, Germany). Photoluminescence (PL) spectra were recorded on a PerkinElmer spectrofluorometer LS 55 (PerkinElmer, Singapore, Singapore). The cells were imaged under LSM7 DUO confocal laser scanning microscope (CLSM; Carl Zeiss Microscopy, Jena, Germany) and fluorescence microscope (IX71, Olympus, Tokyo, Japan). The absorbance was measured using a spectrophotometer (Thermo Fisher Scientific, Waltham, MA, USA).

### 2.2. Synthesis of 1-(2-Hydroxyethyl)-4-methylpyridinium Bromide (Compound 1)

An acetonitrile (20 mL) solution of 4-methylpyridine (0.93 g, 10.00 mmol) had 2-bromoethanol (2.5 g, 20.00 mmol) added to it. The mixture was stirred at reflux temperature (82 °C) under nitrogen for 24 h and then cooled to room temperature. Then, the reaction mixture was precipitated in ethyl acetate (EA) (200 mL). The solid residue was filtrated and washed with excess EA. The filtrate was collected and dried in a vacuum oven. Compound 1 was a light brown powder in 86% yield. ^1^H NMR (400 MHz, DMSO-*d6*, δ): 8.91 (d, 2H, *J* = 6.8 Hz), 7.99 (d, 2H, *J* = 6.4 Hz), 4.64 (t, 2H, *J* = 15.6 Hz), 3.81 (t, 2H, *J* = 15.6 Hz), 2.60 (s, 3H). ^13^C NMR (100 MHz, DMSO-*d6*) δ: 158.5, 143.9, 127.7, 61.9, 59.7, 21.2. HRMS (LDI-TOF) m/z calcd for [C_8_H_12_NO]^+^, 138.0919 [M-Br]+; found, 138.0920 [M-Br]+.

### 2.3. 1-[2-Hydroxyethyl]-4-[4-(1,2,2-triphenylvinyl)styryl]pyridinium Bromide (TPE-Py-OH)

A solution of 1 (0.5 g, 2 mmol) and 4-(1,2,2-triphenylvinyl)benzaldehyde (1.01 g, 2.8 mmol) was refluxed at temperature (78 °C) under nitrogen in dry ethanol (20 mL) catalyzed by three drops of piperidine. After cooling to ambient temperature, the solvent was evaporated under reduced pressure. The residue was purified by a silica gel column chromatography (50 mesh) using dichloromethane/methanol mixture (5:1 *v*/*v*) as eluent to obtain TPE-Py-OH as a yellow solid in 64% yield (0.72 g, 1.29 mmol). ^1^H NMR (400 MHz, DMSO-*d6*) δ: 8.82 (d, 2H, *J* = 6.8 Hz), 8.16 (d, 2H, *J* = 6.8 Hz), 7.88 (d, 1H, *J* = 16.0 Hz), 7.50 (d, 2H, *J* = 8.4 Hz), 7.42 (d, 1H, *J* = 16.4 Hz), 7.16–6.94 (m, 17H), 4.51 (t, 2H, *J* = 10.0 Hz), 3.82 (m, 2H), 3.5(brs, 1H). ^13^C NMR (100 MHz, DMSO-*d6*) δ: 152.70, 145.35, 144.50, 142.79, 142.71, 142.56, 141.40, 140.07, 139.72, 133.10, 131.25, 130.53, 130.51, 130.43, 127.79, 127.68, 127.51, 126.69, 126.62, 123.22, 123.02, 61.92, 59.83. HRMS (LDI-TOF) *m*/*z* calcd for [C_15_H_30_NO]^+^, 480.2327 [M-Br]+; found, 480.2317 [M-Br]+.

### 2.4. Cellular Uptake and Subcellular Distribution

HepG2 human HCC cells were cultured in DMEM medium supplemented with 10% FBS and 1% penicillin-streptomycin at 37 °C in a 5% CO_2_, 90% relative humidity incubator. For cell imaging, the HepG2 cells were seeded in slide chambers at a density of 10^5^ and cultured overnight for adhesion. Then, cells were incubated with TPE-Py-OH at various concentrations (2, 5, and 10 μM) for 30 min and continued to incubate with fresh medium for 12 h. Nuclei were labeled with DAPI (1 μg/mL) overnight. After rinsing with PBS, the cells were imaged under a confocal laser scanning microscope (CLSM, Nikon Corporation, Tokyo, Japan). To further explore the subcellular distribution of TPE-Py-OH, 2 × 10^5^ HepG2 cells were plated in the 35-mm confocal dish and cultured overnight, then incubated with 5 μM TPE-Py-OH for 30 min. After rising with PBS, the cells were then stained with 50 nM MitoTracker Red FM, or continuously incubated with fresh culture medium and then stained with 1 μg/mL BODIPY for 30 min, respectively. After three washes, the cell images were captured under CLSM, and the data was analyzed by NIS-Elements Imaging Software (Nikon Corporation, Tokyo, Japan) and Image-J (National Institutes of Health freeware, Bethesda, MD, USA). For TPE-Py-OH: λex = 405 nm and band-pass filter λ = 550–600 nm. For DAPI: λex = 405 nm and band-pass filter λ = 425–475 nm. For MitoTracker Red: λex = 581 nm and band-pass filter λ = 600–650 nm. For BODIPY 493/503: λex = 488 nm and band-pass filter λ = 500–550 nm.

### 2.5. Cytotoxicity Studies In Vitro

Cell viability after incubation with various concentrations of TPE-Py-OH was evaluated by CCK8 assays. Briefly, the cells at a density of 1 × 10^4^ were seeded into a 96-well plate overnight and then incubated with a series of concentrations of TPE-Py-OH (0, 1, 3, 5, 7, 10, and 15 μM). After pre-treatment, all the cells were incubated with 100 μL fresh medium including with 10 μL CCK8 solution per well and then continued to incubate for 4 h. The absorbance of the solution at 450 nm was measured using a multimode plate reader (Perkin Elmer Pte. Ltd., Singapore, Singapore).

For cytotoxicity effect induced by PDT, the cells were incubated with various concentrations of TPE-Py-OH for 30 min and continued to incubate with fresh medium for 12 h and then exposed to blue laser irradiation with different duration (450 nm, 30 mW/cm^2^, 1.8 J/cm^2^–18 J/cm^2^). The cells were as negative controls without both laser irradiation and TPE-Py-OH treatment. Sequentially, the cells continued to incubate for 24 h and the cell viability was measured according to the above description.

### 2.6. Intracellular ROS Detection

Intracellular ROS production was measured by 2,7-dichlorodihydrofluorescein diacetate (DCFH-DA) probe according to the manufacturer’s instructions. After incubation in the presence or absence of TPE-Py-OH for 30 min, the cells were loaded with 10 μM DCFH-DA at 37 °C for 30 min in dark. The cells were replaced with fresh medium and then exposed to 450 nm blue laser irradiation at the power density of 30 mW/cm^2^, then immediately imaged under a fluorescence microscope (IX71, Olympus, Tokyo, Japan). For DCFH-DA: λex = 488 nm and band-pass filter λ = 500–550 nm.

### 2.7. Calcein-AM and Propidium Iodine (PI) Staining Assay

The live and dead cells were identified using a calcein/PI double stain kit according to the protocol. After treatment, the cells were incubated with 2 μM calcein-AM and 5 μM PI for 30 min at 37 °C. The fluorescence images were captured under a fluorescence microscope (IX71, Olympus, Tokyo, Japan).

### 2.8. Flow Cytometry Analysis

The apoptotic and necrotic cells were detected by Annexin V-FITC/PI dual staining using flow cytometry. The cells were seeded into a 24-well plate overnight and then incubated with TPE-Py-OH for 30 min. After exposure to laser irradiation (450 nm, 30 mW/cm^2^, 4 min, or 8 min), the cells were further incubated for 24 h, then collected and resuspended in 100 μL of binding buffer. According to the manufacturer’s instructions, the cells were incubated with 5 μL Annexin V-FITC and 10 μL PI for 15 min in the dark at room temperature and then immediately measured by flow cytometry (BD Falcon, San Jose, CA, USA).

### 2.9. Mitochondrial Membrane Potential (Δψm) Measurement

The change of mitochondrial membrane potential (Δψm) was measured by JC-1 Dye according to the manufacturer’s instructions. Briefly, the cells were incubated with 2 μg/mL of JC-1 for 20 min at 37 °C after different concentrations of TPE-Py-OH administration and different laser irradiation times, washed twice with PBS, and then imaged under CLSM. For JC-1 (monomer): λex = 488 nm and band-pass filter λ = 500–530 nm; (J-aggregate): λex = 585 nm and band-pass filter λ = 590 nm.

### 2.10. Long-Term Cell Tracking and In Vitro PDT

The cells were first stained with 10 μM TPE-Py-OH for 12 h and then rinsed with PBS to remove the extracellular molecules. The image was captured as a reference. The cells were incubated continuously and one-third of them were sub-cultured into another dish for continual tracking every 2 days. Other cells were seeded into the 35-mm confocal dish. After attachment for 24 h, the fluorescence of TPE-Py-OH in cells was imaged by CLSM, the ROS level in cells was performed according to the above description. The step was repeated every 2 days until non-fluorescence in cells.

To confirm the long-term localization of intracellular TPE-Py-OH, the HepG2 cells were incubated with 10 μM TPE-Py-OH for 12 h and then cultured with fresh DMEM medium, the daughter cells were sequentially co-stained with 1 μg/mL BODIPY for 30 min on days 1, 3, 5, and 7 and their fluorescence images were captured under a fluorescence microscope (IX71, Olympus, Tokyo, Japan).

For long-term PDT in vitro, the HepG2 cells were incubated with 10 μM TPE-Py-OH for 12 h and replaced with fresh medium for following experiments. On days 1, 3, 5, and 7 after TPE-Py-OH treatment, the cells were seeded into 35-mm confocal dishes. After attachment, the cells were exposed to blue laser irradiation with different duration (450 nm, 30 mW/cm^2^, 18–45 J/cm^2^) with half of the confocal dish covered by foil paper to avoid light irradiation. Then the calcein/PI double stain was used to detect the effect of PDT, and the JC-1 mitochondrial membrane potential probe was used for mitochondrial membrane potential measurement. The fluorescence images were captured under a fluorescence microscope (IX71, Olympus, Tokyo, Japan).

### 2.11. Animal Model

Male BALB/c mice (4 weeks old, 20 g) were purchased from HFK Bioscience Co, Ltd. (Beijing, China). All animal experiments were approved by the Ethics Committee of the Union Hospital of Huazhong University of Science and Technology and conducted in accordance with the guidelines of the Department of Laboratory Animals of Tongji Medical College. H22 cells (5 × 10^5^) were subcutaneous injection into the right axilla of each mouse to establish xenograft liver tumor model, and the tumor volume reached 60 mm^3^ after 5 days.

### 2.12. In Vivo Multiple Light-Activated PDT

Tumor-bearing mice were randomized into 5 groups of 8 animals per group: (Group 1: TPE-Py-OH 3 IR) 50 ug/100 uL TPE-Py-OH injected intratumorally, and then underwent laser irradiation (450 nm, 100 mw/cm^2^, 10 min; 60 J/cm^2^) on days 1, 3, and 5 after injection; (Group 2: TPE-Py-OH 1 IR) 50 ug/100 uL TPE-Py-OH injected intratumorally, and then underwent laser irradiation (450nm, 100 mw/cm^2^, 10min; 60 J/cm^2^) on the first day after injection; (Group 3: TPE-Py-OH non IR) 50 ug/100 uL TPE-Py-OH injected intratumorally without irradiation; (Group 4: 3 IR only) 100 μL of PBS injected intratumorally, and then underwent laser irradiation (450 nm, 100 mw/cm^2^, 10 min; 60 J/cm^2^) on days 1, 3, and 5 after injection; (Group 5: NC) negative control.

Seven days after injection, 3 mice of every group were sacrificed. Tumors were collected and kept in 4% formalin followed by embedded in paraffin. Slices cut from the paraffin sections were stained by hematoxylin and eosin (H&E) and TUNEL before being scanned with a fluorescence microscope (IX71, Olympus, Tokyo, Japan). The tumor size was measured by a caliper every 2 days and calculated as the volume = (tumor length) × (tumor width)^2^ × 0.5.

## 3. Results and Discussions

### 3.1. Synthesis and Characterization

**Figure 1:**
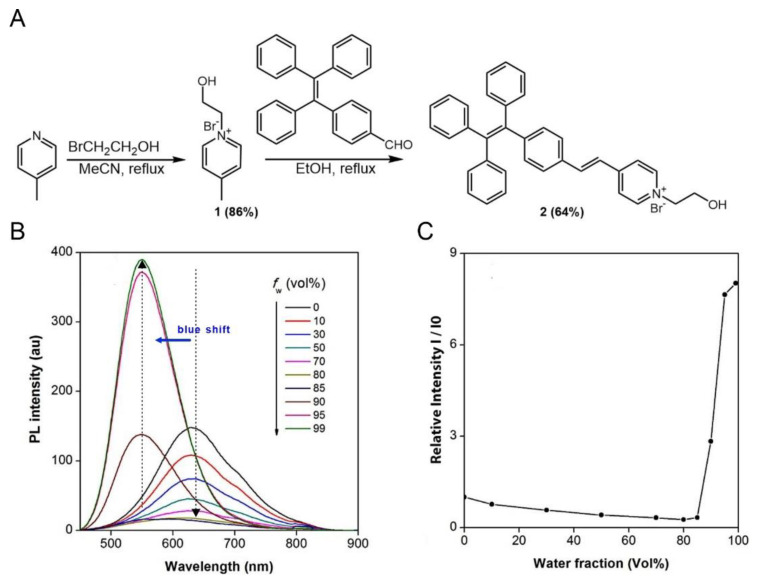
(**A**) Synthesis of molecule 2 (TPE-Py-OH). (**B**) PL spectra of TPE-Py-OH in DMSO/H_2_O mixtures with different water fractions (fw). Concentration: 100 µM; excitation wavelength: 405 nm. (**C**) Plot of PL intensity versus the composition of the DMSO/H_2_O mixtures of TPE-Py-OH.

TPE-Py-OH was synthesized according to the synthetic route shown in Figure 1A. Compound 1 was obtained by quaternization of 4-methylpyridine with 2-bromoethanol. The product was purified by precipitation of the reaction mixture in excess EA. The product was obtained by filtrating and washing with EA, which was suitable to be carried forward. 4-(1,2,2-Triphenylvinyl)benzaldehyde (TPE-CHO) was synthesized according to the reported method [46,47]. Finally, compound 1 and TPE-CHO were refluxed in ethanol to give TPE-Py-OH in reasonable yield by aldol condensation. Unexpectedly, TPE-Py-OH can be purified with silica column by using high polarity solvent mixture (DCM: MeOH = 5:1) as eluent. Compound 1 and TPE-Py-OH were fully characterized by HRMS, and ^1^H and ^13^C NMR spectroscopies, and gave satisfactory analysis results corresponding to their chemical structures (Figures S1–S4). Due to its ionic character, TPE-Py-OH has good solubility in most of organic solvents like THF, DCM, and DMSO, but it is not soluble in water.

### 3.2. Aggregation and Micellization of TPE-Py-OH

Figure S5 shows the UV spectrum of TPE-Py-OH (10 µM) in DMSO solutions. The maximum absorption peak of TPE-Py-OH is located at 395 nm. For convenient bio-application, 405 nm was utilized as excitation wavelength for PL measurement. Photoexcitation of its DMSO solution (100 µM) induces a red emission at 650 nm (Figure 1B,C), giving a large Stokes shift of 9932 cm^−1^ due to its extended conjugation as well as the intramolecular charge transfer (ICT) effect from the electron-donating TPE moiety to the electron-accepting pyridinium unit [47]. Due to the positively charged characteristics and pendant hydroxy group, TPE-Py-OH is amphiphilic and shows reversible aggregation behaviors in DMSO/H_2_O mixture (Figure S6A). Normally, solutions with a concentration of 10^−5^–10^−6^ M are used in PL measurements. A highly concentrated solution (100 µM) of TPE-Py-OH is employed for the analysis because TPE-Py-OH only forms aggregates or micelles at higher concentrations. According to previous studies, the aromatic rings on AIEgens have larger twisted angles in the aggregated state than in the solution state [23]. Thus, the AIEgens generally show blue-shifted in the aggregated state compared to that in the solution state. As shown in Figure 1B, TPE-Py-OH emits strongly in aqueous solution at 565 nm due to the activation of the AIE effect by micelles formation. The emission becomes weaker and shifts to 650 nm upon addition of a small amount of DMSO, presumably owing to the disintegration of the micelles, which releases free molecules with a more planar conformation to the solution [48].

To study the micellization behaviors of TPE-Py-OH, the PL of its solutions at different concentrations were investigated by taking advantage of its AIE property. The PL intensity of TPE-Py-OH increases with increasing the solution concentration with stable emission spectra (Figure S6B). The plot of relative PL intensity (*I*/*I*_0_) against the logarithm solution concentration generates two lines, the intersection of which determines the critical micelle concentration (CMC) and is found to be 0.3 mM in H_2_O/DMSO mixtures (*v*/*v* 90%). TPE-Py-OH is molecularly dispersed in solutions with concentrations of below 0.3 mM and displayed weak emission. When the concentration of TPE-Py-OH was more than or equal to 0.3 mM, the FL intensity was remarkably increased due to the AIE feature.

### 3.3. Dual-Organelles Targeting and Cell Imaging of TPE-Py-OH

We next studied the cellular uptake of TPE-Py-OH in HepG2 cells. The cells were treated with TPE-Py-OH and then observed by CLSM upon excitation at 405 nm with a collection of fluorescent signals above 585 nm. As shown in Figure S7, the bright yellow fluorescence could be detected from the cytoplasm after incubation with TPE-Py-OH for 30 min, which demonstrated that TPE-Py-OH could be internalized with high efficiency by HepG2 cells. Meanwhile, the fluorescence structure in cells was mainly in funicular shape when TPE-Py-OH entered into cells in short time and then fluorescence in punctate shape gradually enhanced, which demonstrated the aggregation of free TPE-Py-OH molecules in cytoplasm as the incubation time prolonged. This phenomenon indicated that the organelles targeting of TPE-Py-OH maybe occur in live cells.

**Figure 2:**
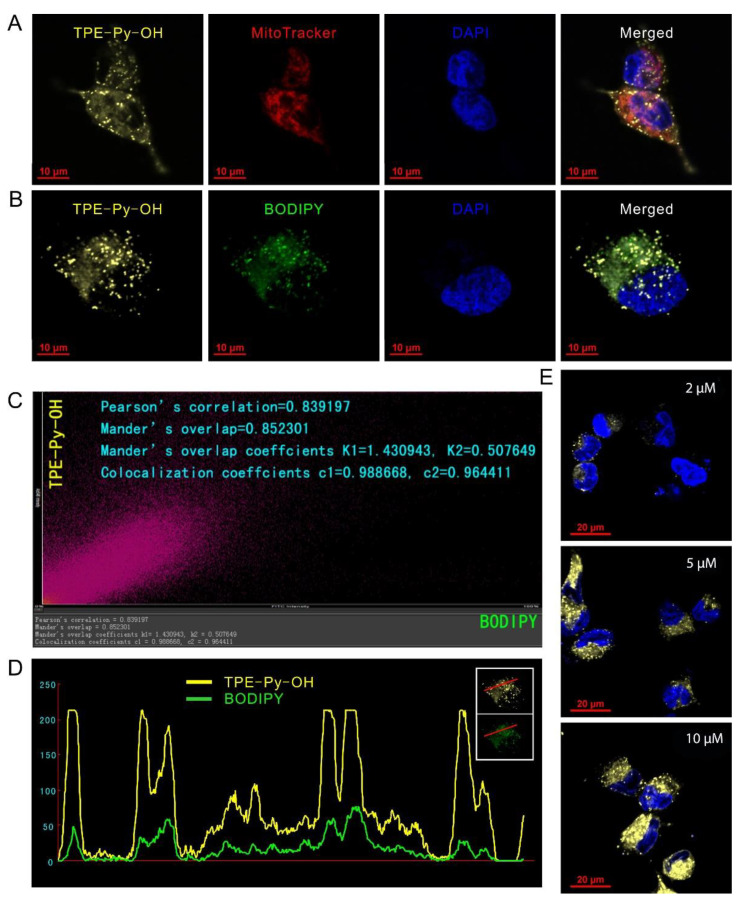
Fluorescence imaging of TPE-Py-OH in HepG2 cells. (**A**) Co-staining of 5 μM TPE-Py-OH (λex = 405 nm) and 50 nM MitoTracker Red (λex = 581 nm) for 30 min. (**B**) Co-staining of cells incubated with 5 μM TPE-Py-OH for 30 min, followed by continuous incubation in fresh medium for 12 h and then staining with 1 μg/L BODIPY (λex = 488 nm) for 30 min. (**C**,**D**) Quantitation analysis of the cells co-stained with TPE-Py-OH and BODIPY. (**E**) Images of TPE-Py-OH in HepG2 cells incubated at various concentrations for 30 min.

To explore the subcellular location of TPE-Py-OH in living cells, HepG2 cells were co-stained with TPE-Py-OH and commercial fluorescent probes of mitochondria (MitoTracker Red) and LDs (BODIPY 493/503), respectively. As shown in Figure 2A, a large amount of yellow fluorescence from TPE-Py-OH merged with red fluorescence from MitoTracker Red in cells. However, some extra bright yellow fluorescence signals with punctate shape did not overlay the punctate red mitochondrial fluorescence. Sequentially, the co-localization experiment with BODIPY showed that the yellow punctate shape fluorescence from TPE-Py-OH and green fluorescence from BODIPY were co-localized well in live cells (Figure 2B). The Pearson’s correlation was further quantitatively determined, which is high as 0.839 (Figure 2C). The intensity profile for the region of interest line across the cells also varied in close synchrony (Figure 2D). All the results indicated that TPE-Py-OH has a dual-targeting ability of mitochondria and LDs with high selectivity due to their structures of membrane phospholipid. Furthermore, it seems that the more dissociative TPE-Py-OH in cytoplasm could be finally inclined to LDs, and then stored in these organelles and kept stable in cells. Additionally, the fluorescent intensity of TPE-Py-OH in cells was dependent on its concentration in culture medium, which demonstrated that stronger fluorescence was captured in cells incubated with higher concentration (Figure 2E).

### 3.4. In Vitro Anti-Cancer Efficacy of TPE-Py-OH

**Figure 3:**
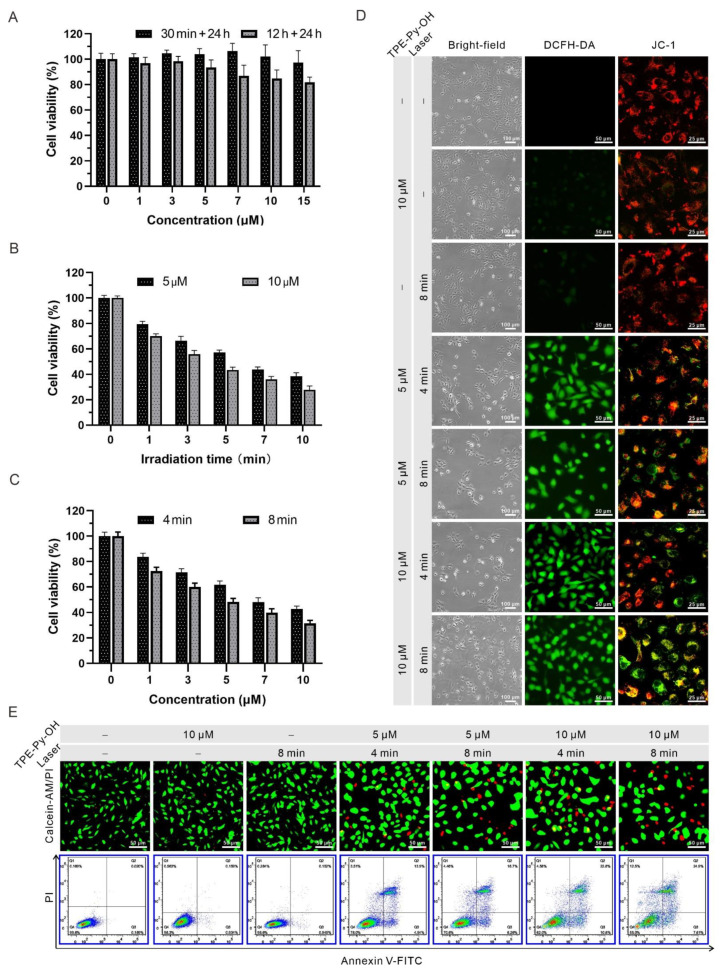
In vitro ROS generation and anti-tumor efficacy of TPE-Py-OH. (**A**) The viability of HepG2 cells pretreated with different concentrations of TPE-Py-OH for different times without light irradiation. (**B**) The viability of HepG2 cells pretreated with TPE-Py-OH, followed by different irradiation times (450 nm, 30 mW/cm^2^). (**C**) The viability of HepG2 cells pretreated with different concentrations of TPE-Py-OH, followed by 4 min or 8 min irradiation (450 nm, 30 mW/cm^2^; 7.2 J/cm^2^ or 14.4 J/cm^2^). (**D**) Detection cell morphology, ROS generation, and mitochondrial membrane potential (ΔΨm) after different treatment of HepG2 cells. For DCFH-DA: λex = 488 nm and band-pass filter λ = 500–550 nm. For JC-1 (monomer): λex = 488 nm and band-pass filter λ = 500–530 nm; (J-aggregate): λex = 585 nm and band-pass filter λ = 590 nm. (**E**) Fluorescence images stained with calcein-AM/PI (For calcein-AM λex = 490 nm, For PI λex = 545 nm) and flow cytometry stain with Annexin V-FITC/PI after different treatment of HepG2 cells. For flow cytometry: Q1: Necrotic cells; Q2: Late apoptotic cells; Q3: Early apoptotic cells; Q4: Normal cells.

Biocompatibility is a vital parameter of the PS for cancer therapy in vivo, and thus the biocompatibility of TPE-Py-OH on HepG2 cells was assessed via CCK8 assay before the in vitro PDT experiments. The cytotoxicity of TPE-Py-OH with different concentrations upon incubation with HepG2 cells in dark condition was first evaluated. As shown in Figure 3A, no significant change was observed in cell viability at 24 h after 30 min incubation without light irradiation. Even after 12 h incubation with 15 µM TPE-Py-OH, the cell viability was more than 80%, which indicated minimal cytotoxicity of TPE-Py-OH to cells and excellent biocompatibility. Then the PDT anti-cancer efficiency of TPE-Py-OH against HepG2 cells was further assessed. As shown in Figure 3B,C, the cell viability was significantly decreased in the presence of 450 nm blue laser irradiation. Meanwhile, the amount of cell death was associated with the light duration and the incubation concentration of TPE-Py-OH. The process of ROS generation and mitochondrial-reliant photodynamic killing of TPE-Py-OH was illustrated in Figure 3D. The intracellular ROS level after PDT treatment was measured via ROS indicator 2,7-dichlorodihydrofluorescein diacetate (DCFH-DA), which is highly fluorescent in the presence of ROS in living cells. A JC-1 dye was applied to monitor the change of mitochondrial membrane potential (Δψm). The decline of relative intensity of red to green fluorescence of JC-1 dye could indicate the depolarization of mitochondria, which is commonly used as an indicator of mitochondrial damage [49]. Additionally, after incubation with TPE-Py-OH followed by light irradiation, the appearance change of HepG2 cells was observed. As the concentration of TPE-Py-OH and irradiation time increased, HepG2 cells gradually shrank and turned round, indicating early apoptosis. After staining with DCFH-DA, green fluorescence was negligible without light irradiation or TPE-Py-OH. In contrast, cells incubated with TPE-Py-OH showed bright green fluorescence with light irradiation, which indicated significantly increased ROS production. The results of JC-1 imaging showed that TPE-Py-OH could efficiently promote the decline of Δψm under light irradiation and the decline of Δψm was concentration- and irradiation time-dependent. To further explore the PDT effect of TPE-Py-OH, HepG2 cells were stained with calcein-AM (green fluorescence) and PI (red fluorescence) to label live and dead cells (Figure 3E). The increased red fluorescence in TPE-Py-OH plus light irradiation group indicated good photodynamic cell death effect. An Annexin V-FITC/PI apoptosis detection kit was used to further investigate the cell death modality induced by PDT treatment (Figure 3E). An increased number of both necrotic cells and apoptotic cells were observed with an elevation of the TPE-Py-OH concentration and irradiation time. All of the above results indicated that TPE-Py-OH could induce cell death through ROS generation and mitochondrial damage in the presence of light irradiation, and the dominant cell death is closely related to the light duration and incubation concentration.

### 3.5. In Vitro Long-Term Tracking and PDT of TPE-Py-OH

**Figure 4:**
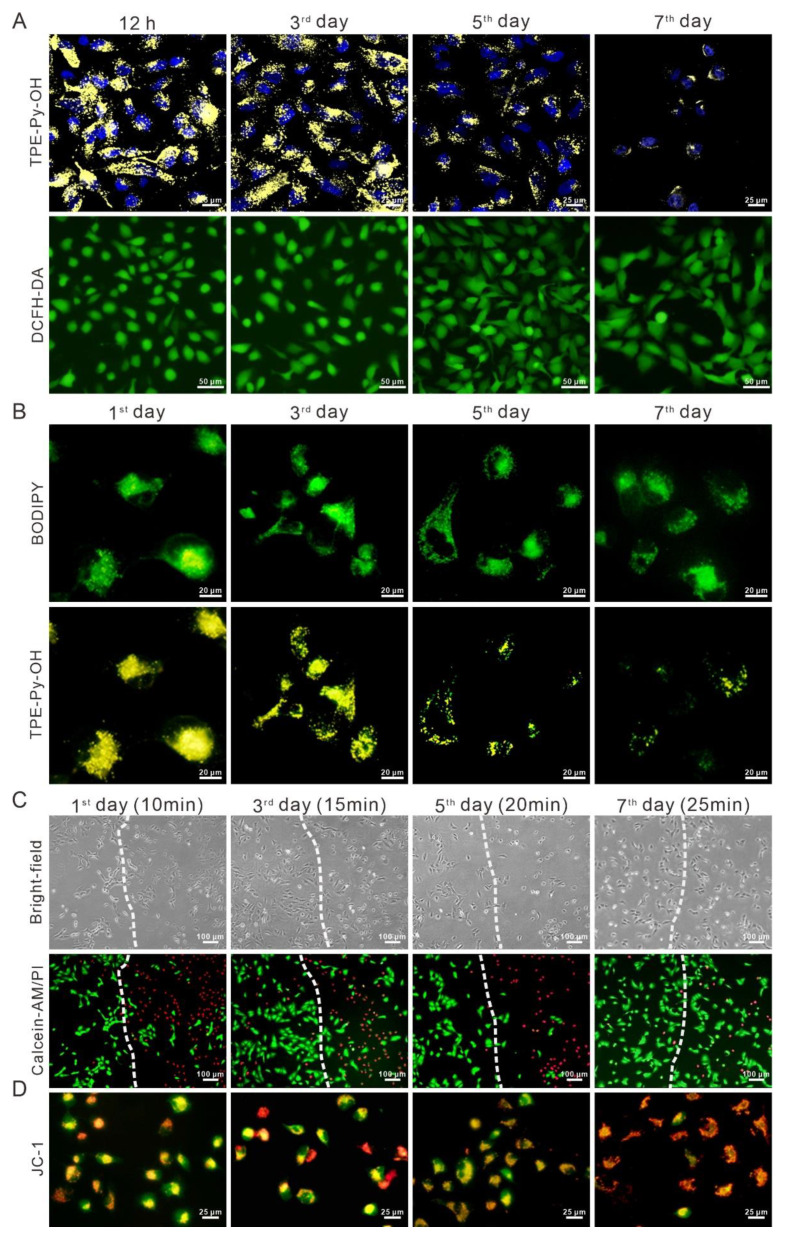
Long-term tracking and PDT effect of HepG2 cells stained with 10 μM TPE-Py-OH for 12 h and continuously cultured for different time. (**A**) Fluorescent images of TPE-Py-OH and intracellular ROS generation after light irradiation (450 nm, 30 mW/cm^2^, 10 min). (**B**) Co-staining of HepG2 cells stained with TPE-Py-OH and continuously cultured for different time, followed by staining with 1 μg/mL BODIPY for 30 min. (**C**) Fluorescence images stained with calcein-AM/PI of HepG2 cells cultured for different time. left part of the dotted line: without light irradiation, right part of the dotted line: with light irradiation. (**D**) Fluorescence images stained with JC-1 of HepG2 cells cultured for different time with irradiation.

To investigate the long-term retention of TPE-Py-OH in living cells, the HepG2 cells stained with TPE-Py-OH were continuously cultured and their fluorescent signal was monitored under CLSM. As shown in Figure 4A, the yellow fluorescence from TPE-Py-OH was recorded after 12 h of incubation as a reference. Sequentially, the fluorescence signal was captured every 2 days, and the intracellular yellow fluorescence still existed even on the seventh day after incubation with TPE-Py-OH, which demonstrated the long-term intracellular retention ability of TPE-Py-OH. Although the fluorescence intensity decreased as cell divided due to the reduction of the number of the AIEgen in each cell, the residual PSs could still be activated by light irradiation and induce ROS generation, which was confirmed by the DCFH-DA staining. Since previous result showed intracellular storage of TPE-Py-OH, we then explored the location of long-term retention of TPE-Py-OH in live HepG2 cells. Although TPE-Py-OH decreased in single cell as cell division, the yellow fluorescence was still co-localized with BODIPY, indicating that they could keep staying in LDs with high binding characteristics in Figure 4B. To verify long-term photodynamic effect of the remaining TPE-Py-OH in cells, the PDT efficiency was investigated using calcein-AM/PI dual staining. As shown in Figure 4C, the region of light irradiation (right part of the dotted line) showed increased red fluorescence of PI stain even on the seventh day after treatment with TPE-Py-OH in contrast with the region without light irradiation (left part of the dotted line). In the region of light irradiation, cell morphologic changes of the early stage of apoptosis were also observed in the bright field under optical microscope. Although the efficiency of PDT was decreased with the passage of time, the obvious photodynamic killing effect could last for at least 1 week after primary TPE-Py-OH treated with prolonged light irradiation. It seems that TPE-Py-OH can combine with and store in intracellular LDs with stable fluorescence and photodynamic tumor-killing effect. Previous results also showed that TPE-Py-OH could induce mitochondrial damage and dysfunction due to its ability to target mitochondria. Since the TPE-Py-OH could aggregate in LDs after long-term incubation, we further explored the change of damage mechanism. Interestingly, JC-1 staining demonstrated that TPE-Py-OH could still induce dramatically mitochondrial damage in spite of tending to accumulate into LDs in Figure 4D. Singlet oxygen species produced by PDT can oxidize intracellular LDs to form lipid oxide or lipid peroxide [50]. The intracellular lipid peroxidation could initiate cellular ferroptosis, which can induce mitochondrial morphology disruption and depolarization in this process [51]. This may be the mitochondrial damage mechanism of TPE-Py-OH stored in LDs. Hence, the TPE-Py-OH could store in LDs in a relatively stable state and maintain its ROS generation ability for more than 7 days, which allowed multiple-light activated PDT effect and mitochondrial damage.

### 3.6. In Vivo Multiple Light-Activated PDT Effect of TPE-Py-OH

**Figure 5:**
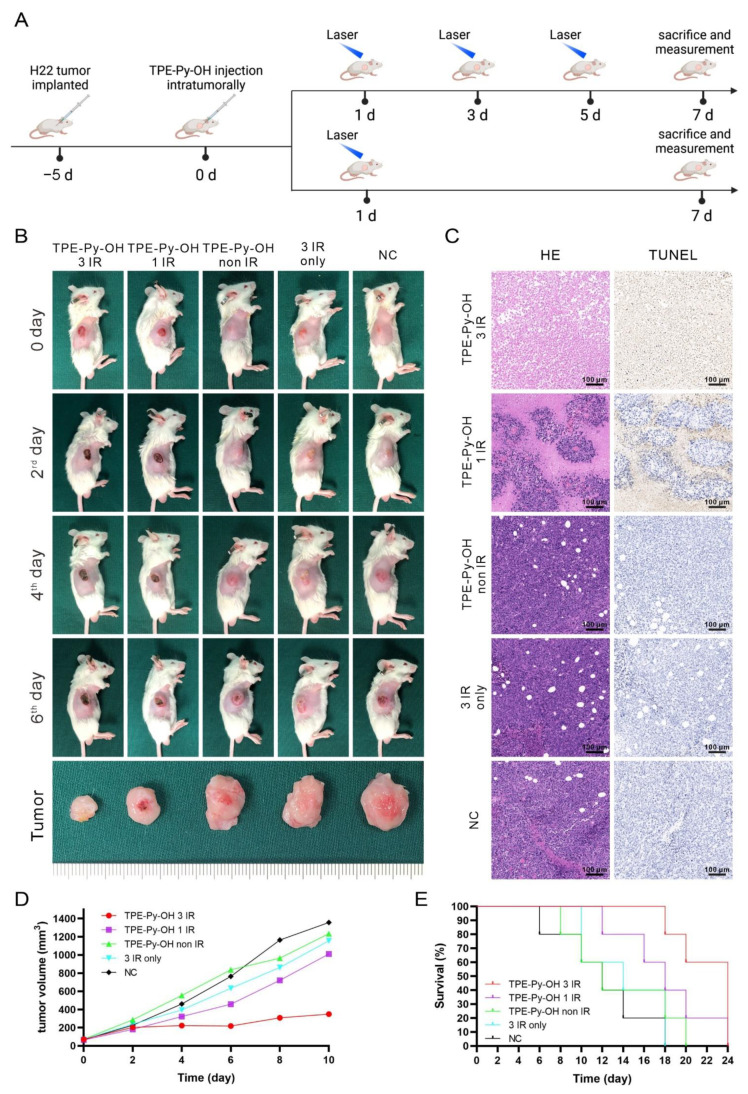
In vivo multiple light-activated PDT effect of TPE-Py-OH in H22 tumor-bearded mice. (**A**) Scheme of in vivo PDT experiment. (**B**) Representative photographs of mice with treatments at different days, the irradiation was performed with 450 nm laser (100 mW/cm^2^) for 10 min. (**C**) H&E stained and TUNEL stained tumor tissue slices after 7 days of treatments. (**D**) Tumor growth curves of mice with the various treatments. (**E**) Survival of mice with the various treatments.

To further evaluate the potential of PDT performance of TPE-Py-OH in vivo, we carried out a mice H22 tumor model. The tumor model was established by subcutaneous injection of 5 × 10^5^ H22 cells in BALB/c mice. After the tumors had become 60 mm^3^ in size, TPE-Py-OH were injected intratumorally. In vivo distribution of TPE-Py-OH in mice over time was monitored by whole animal fluorescence. As shown in Figure S8A, local fluorescence still existed even at 12th day after TPE-Py-OH administration. The compression splice of tumor tissue shows bright yellow fluorescence in tumor cells (Figure S8B). Based on the long-term retention of TPE-Py-OH in tumor foci after administration, we hypothesized that the remaining TPE-Py-OH could kill the tumor cells by repeated irradiation after one administration via PDT effect. Subsequently, to explore the multiple light-activated PDT performance of TPE-Py-OH, H22 tumor-bearing mice were randomized into five groups: (1) TPE-Py-OH with three irradiations; (2) TPE-Py-OH with one irradiation; (3) TPE-Py-OH without irradiation; (4) PBS with irradiation; (5) negative control. TPE-Py-OH was intratumorally injected and further irradiated with laser (450 nm, 100 mW/cm^2^, 10 min) and the course of treatment is shown in Figure 5A. As shown in Figure 5B, the mice injected TPE-Py-OH with laser irradiation three times exhibited larger scale local necrosis of tumor and better tumor inhibition than the group treated with irradiation one time. TPE-Py-OH without irradiation group, PBS with irradiation group and negative control group were found to exhibit negligible tumor necrosis and inhibition. The tumor tissues were harvested after the mice were sacrificed on the seventh Day. Hemotoxylin/eosin (H&E) staining of tumor tissue showed a majority of dead cells in TPE-Py-OH with the three-time irradiation group and relative mild tumor cells death in TPE-Py-OH with the one-time irradiation group (Figure 5C). A high apoptosis rate was also observed by TUNEL stain, which demonstrated the multiple light-activated PDT effect of TPE-Py-OH after once injection for more than 1 week. This result was consistent with previous in vitro experiments. Tumor growth in the different groups was monitored by recording the tumor volume every 2 days for a period of 10 days. The tumor volume of TPE-Py-OH with three-time irradiation group was obviously smaller than other groups (Figure 5D). Although TPE-Py-OH with the one-irradiation group showed mild tumor inhibition after treatment, the tumor volume significantly increased after treatment ended. Mice survival was monitored, as shown in Figure 5E. Mice treated with TPE-Py-OH and irradiation three times showed better survival than other groups. All these results demonstrate that TPE-Py-OH could accumulate in the tumor region for a long time and provide multiple light-activated PDT effect for a long time.

## 4. Conclusions

In summary, we have developed a dual-organelles-targeting AIEgen based on tetraphenylethylene derivative (TPE-Py-OH). TPE-Py-OH can target both mitochondria and LDs, exhibits excellent ability of long-term retention, has stable fluorescence emission and multiple light-activated PDT effect in cancer cells. By storing in intracellular LDs, the fluorescence of TPE-Py-OH in cells can be maintained for a long time, and the photodynamic tumor-killing effect could still be activated by light irradiation at 7 days after a single treatment with TPE-Py-OH in vitro and in vivo. We believe that this AIEgen will be promising for the long-term tracking and multiple light-activated photodynamic ablation of HCC with sustained effectiveness. Although the tumor-targeting ability and the approach of drug administration is less than perfect, the hydroxy group (-OH) of this AIEgen provides excellent molecular modifiability and carrier combinability. Further modification of molecular structure and combination with nanocarrier could improve the cancer selectivity and fluorescence properties of TPE-Py-OH. Based on molecular characteristics of TPE-Py-OH, new PSs with improved function and a better administration approach can be designed to achieve multiple light-activated PDT.

## Data Availability

Data sharing not applicable.

## Supplementary Materials

The following are available online at https://www.mdpi.com/article/10.3390/pharmaceutics14020459/s1, Figure S1: HRMS spectra of molecule 1; Figure S2: HRMS spectra of TPE-Py-OH; Figure S3: ^1^H NMR spectra of (A) TPE-Py-OH and (B) molecular 1 in DMSO-*d*_6_; Figure S4: ^13^C NMR spectra of (A) TPE-Py-OH and (B) molecular 1 in DMSO-*d*_6_; Figure S5: UV spectra of TPE-Py-OH at the concentration of 10 μM in DMSO; Figure S6: (A) PL spectra of TPE-Py-OH in H_2_O/DMSO mixtures (*v*/*v* 90%) with different concentrations. Excitation wavelength: 405 nm. (B) Plot of PL intensity versus the concentrations of TPE-Py-OH. Inset: Photograph of TPE-Py-OH with various concentrations taken under 365 nm UV irradiation; Figure S7: The dynamic monitoring of the distribution of TPE-Py-OH in living cells. The HepG2 cells were incubated with 5 μM TPE-Py-OH for 30 min and then replaced with fresh medium, Figure S8: (A) The distribution of TPE-Py-OH in vivo after intratumoral injection over time. (B) Imaging of compression slice of H22 tumor on day 12 after TPE-Py-OH administration.

## References

[1] Vogel A., Cervantes A., Chau I., Daniele B., Llovet J.M., Meyer T., Nault J.C., Neumann U., Ricke J., Sangro B. (2018). Hepatocellular carcinoma: ESMO Clinical Practice Guidelines for diagnosis, treatment and follow-up. Ann. Oncol..

[2] Yang G., Xiong Y., Sun J., Wang G., Li W., Tang T., Li J. (2020). The efficacy of microwave ablation versus liver resection in the treatment of hepatocellular carcinoma and liver metastases: A systematic review and meta-analysis. Int. J. Surg..

[3] Makary M.S., Khandpur U., Cloyd J.M., Mumtaz K., Dowell J.D. (2020). Locoregional Therapy Approaches for Hepatocellular Carcinoma: Recent Advances and Management Strategies. Cancers.

[4] Minami Y., Kudo M. (2021). Image Guidance in Ablation for Hepatocellular Carcinoma: Contrast-Enhanced Ultrasound and Fusion Imaging. Front. Oncol..

[5] Castano A.P., Mroz P., Hamblin M.R. (2006). Photodynamic therapy and anti-tumour immunity. Nat. Rev. Cancer.

[6] Kumar A., Morales O., Mordon S., Delhem N., Boleslawski E. (2021). Could Photodynamic Therapy Be a Promising Therapeutic Modality in Hepatocellular Carcinoma Patients? A Critical Review of Experimental and Clinical Studies. Cancers.

[7] Broekgaarden M., Weijer R., van Gulik T.M., Hamblin M.R., Heger M. (2015). Tumor cell survival pathways activated by photodynamic therapy: A molecular basis for pharmacological inhibition strategies. Cancer Metastasis Rev..

[8] Ma Y., Huang J., Song S., Chen H., Zhang Z. (2016). Cancer-Targeted Nanotheranostics: Recent Advances and Perspectives. Small.

[9] Gonzalez-Carmona M.A., Bolch M., Jansen C., Vogt A., Sampels M., Mohr R.U., van Beekum K., Mahn R., Praktiknjo M., Nattermann J. (2019). Combined photodynamic therapy with systemic chemotherapy for unresectable cholangiocarcinoma. Aliment. Pharmacol. Ther..

[10] Song W., Kuang J., Li C.X., Zhang M., Zheng D., Zeng X., Liu C., Zhang X.Z. (2018). Enhanced Immunotherapy Based on Photodynamic Therapy for Both Primary and Lung Metastasis Tumor Eradication. ACS Nano.

[11] Maeding N., Verwanger T., Krammer B. (2016). Boosting Tumor-Specific Immunity Using PDT. Cancers.

[12] Jiang R., Dai J., Dong X., Wang Q., Meng Z., Guo J., Yu Y., Wang S., Xia F., Zhao Z. (2021). Improving Image-Guided Surgical and Immunological Tumor Treatment Efficacy by Photothermal and Photodynamic Therapies Based on a Multifunctional NIR AIEgen. Adv. Mater..

[13] He J., Yang L., Yi W., Fan W., Wen Y., Miao X., Xiong L. (2017). Combination of Fluorescence-Guided Surgery With Photodynamic Therapy for the Treatment of Cancer. Mol. Imaging.

[14] Akopov A., Rusanov A., Gerasin A., Kazakov N., Urtenova M., Chistyakov I. (2014). Preoperative endobronchial photodynamic therapy improves resectability in initially irresectable (inoperable) locally advanced non small cell lung cancer. Photodiagnosis Photodyn. Ther..

[15] Jia R., Xu H., Wang C., Su L., Jing J., Xu S., Zhou Y., Sun W., Song J., Chen X. (2021). NIR-II emissive AIEgen photosensitizers enable ultrasensitive imaging-guided surgery and phototherapy to fully inhibit orthotopic hepatic tumors. J. Nanobiotechnology.

[16] Feng Z., Birong W., Zhengfeng Z., Siqin W., Chuxing C., Dang S., Min L. (2021). Photodynamic therapy: A next alternative treatment strategy for hepatocellular carcinoma?. World J. Gastrointest. Surg..

[17] Correia J.H., Rodrigues J.A., Pimenta S., Dong T., Yang Z. (2021). Photodynamic Therapy Review: Principles, Photosensitizers, Applications, and Future Directions. Pharmaceutics.

[18] Zhou Z., Song J., Nie L., Chen X. (2016). Reactive oxygen species generating systems meeting challenges of photodynamic cancer therapy. Chem. Soc. Rev..

[19] Agostinis P., Berg K., Cengel K.A., Foster T.H., Girotti A.W., Gollnick S.O., Hahn S.M., Hamblin M.R., Juzeniene A., Kessel D. (2011). Photodynamic therapy of cancer: An update. CA Cancer J. Clin..

[20] Bonnett R. (1995). Photosensitizers of the porphyrin and phthalocyanine series for photodynamic therapy. Chem. Soc. Rev..

[21] Kang M., Zhang Z., Song N., Li M., Sun P., Chen X., Wang D., Tang B.Z. (2020). Aggregation-enhanced theranostics: AIE sparkles in biomedical field. Aggregate.

[22] Luo J., Xie Z., Lam J.W., Cheng L., Chen H., Qiu C., Kwok H.S., Zhan X., Liu Y., Zhu D. (2001). Aggregation-induced emission of 1-methyl-1,2,3,4,5-pentaphenylsilole. Chem. Commun..

[23] Dai J., Wu X., Ding S., Lou X., Xia F., Wang S., Hong Y. (2020). Aggregation-Induced Emission Photosensitizers: From Molecular Design to Photodynamic Therapy. J. Med. Chem..

[24] Gao Y., Zheng Q.C., Xu S.D., Yuan Y.Y., Cheng X., Jiang S., Kenry, Yu Q.H., Song Z.F., Liu B. (2019). Theranostic Nanodots with Aggregation-Induced Emission Characteristic for Targeted and Image-Guided Photodynamic Therapy of Hepatocellular Carcinoma. Theranostics.

[25] He W., Zhang T., Bai H., Kwok R.T.K., Lam J.W.Y., Tang B.Z. (2021). Recent Advances in Aggregation-Induced Emission Materials and Their Biomedical and Healthcare Applications. Adv. Healthc. Mater..

[26] Zhao N., Li P., Zhuang J., Liu Y., Xiao Y., Qin R., Li N. (2019). Aggregation-Induced Emission Luminogens with the Capability of Wide Color Tuning, Mitochondrial and Bacterial Imaging, and Photodynamic Anticancer and Antibacterial Therapy. ACS Appl. Mater. Interfaces.

[27] Qi J., Ou H., Liu Q., Ding D. (2021). Gathering brings strength: How organic aggregates boost disease phototheranostics. Aggregate.

[28] Cai X., Liu B. (2020). Aggregation-Induced Emission: Recent Advances in Materials and Biomedical Applications. Angew. Chem..

[29] Gao M., Tang B.Z. (2017). Aggregation-induced emission probes for cancer theranostics. Drug Discov. Today.

[30] Li M., Gao Y., Yuan Y.Y., Wu Y.Z., Song Z.F., Tang B.Z., Liu B., Zheng Q.C. (2017). One-Step Formulation of Targeted Aggregation-Induced Emission Dots for Image-Guided Photodynamic Therapy of Cholangiocarcinoma. ACS Nano.

[31] Wu W., Shi L., Duan Y., Xu S., Gao X., Zhu X., Liu B. (2021). Metabolizable Photosensitizer with Aggregation-Induced Emission for Photodynamic Therapy. Chem. Mater..

[32] Tavakkoli Yaraki M., Wu M., Middha E., Wu W., Daqiqeh Rezaei S., Liu B., Tan Y.N. (2021). Gold Nanostars-AIE Theranostic Nanodots with Enhanced Fluorescence and Photosensitization Towards Effective Image-Guided Photodynamic Therapy. Nano-Micro Lett..

[33] Liao Y., Wang R., Wang S., Xie Y., Chen H., Huang R., Shao L., Zhu Q., Liu Y. (2021). Highly Efficient Multifunctional Organic Photosensitizer with Aggregation-Induced Emission for In Vivo Bioimaging and Photodynamic Therapy. ACS Appl. Mater. Interfaces.

[34] Kim S., Tachikawa T., Fujitsuka M., Majima T. (2014). Far-red fluorescence probe for monitoring singlet oxygen during photodynamic therapy. J. Am. Chem. Soc..

[35] Feng G., Qin W., Hu Q., Tang B.Z., Liu B. (2015). Cellular and Mitochondrial Dual-Targeted Organic Dots with Aggregation-Induced Emission Characteristics for Image-Guided Photodynamic Therapy. Adv. Healthc. Mater..

[36] Chen S., Huang B., Pei W., Wang L., Xu Y., Niu C. (2020). Mitochondria-Targeting Oxygen-Sufficient Perfluorocarbon Nanoparticles for Imaging-Guided Tumor Phototherapy. Int. J. Nanomed..

[37] Zhang L., Wang J.L., Ba X.X., Hua S.Y., Jiang P., Jiang F.L., Liu Y. (2021). Multifunction in One Molecule: Mitochondrial Imaging and Photothermal & Photodynamic Cytotoxicity of Fast-Response Near-Infrared Fluorescent Probes with Aggregation-Induced Emission Characteristics. ACS Appl. Mater. Interfaces.

[38] Yu K., Pan J., Husamelden E., Zhang H., He Q., Wei Y., Tian M. (2020). Aggregation-induced Emission Based Fluorogens for Mitochondria-targeted Tumor Imaging and Theranostics. Chem. Asian J..

[39] Zhou T., Zhu J.F., Shang D., Chai C.X., Li Y.Z., Sun H.Y., Li Y.Q., Gao M., Li M. (2020). Mitochondria-anchoring and AIE-active photosensitizer for self-monitored cholangiocarcinoma therapy. Mater. Chem. Front..

[40] Zhang W., Huang Y., Chen Y., Zhao E., Hong Y., Chen S., Lam J.W.Y., Chen Y., Hou J., Tang B.Z. (2019). Amphiphilic Tetraphenylethene-Based Pyridinium Salt for Selective Cell-Membrane Imaging and Room-Light-Induced Special Reactive Oxygen Species Generation. ACS Appl. Mater. Interfaces.

[41] Thiam A.R., Beller M. (2017). The why, when and how of lipid droplet diversity. J. Cell Sci..

[42] Cruz A.L.S., Barreto E.A., Fazolini N.P.B., Viola J.P.B., Bozza P.T. (2020). Lipid droplets: Platforms with multiple functions in cancer hallmarks. Cell Death Dis..

[43] Cheng C., Geng F., Cheng X., Guo D. (2018). Lipid metabolism reprogramming and its potential targets in cancer. Cancer Commun..

[44] Tabero A., Garcia-Garrido F., Prieto-Castaneda A., Palao E., Agarrabeitia A.R., Garcia-Moreno I., Villanueva A., de la Moya S., Ortiz M.J. (2020). BODIPYs revealing lipid droplets as valuable targets for photodynamic theragnosis. Chem. Commun..

[45] Olzmann J.A., Carvalho P. (2019). Dynamics and functions of lipid droplets. Nat. Rev. Mol. Cell Biol..

[46] Leung C.W., Hong Y., Chen S., Zhao E., Lam J.W., Tang B.Z. (2013). A photostable AIE luminogen for specific mitochondrial imaging and tracking. J. Am. Chem. Soc..

[47] Zhao N., Li M., Yan Y., Lam J.W.Y., Zhang Y.L., Zhao Y.S., Wong K.S., Tang B.Z. (2013). A tetraphenylethene-substituted pyridinium salt with multiple functionalities: Synthesis, stimuli-responsive emission, optical waveguide and specific mitochondrion imaging. J. Mater. Chem. C.

[48] Chen Y., Li M., Hong Y., Lam J.W., Zheng Q., Tang B.Z. (2014). Dual-modal MRI contrast agent with aggregation-induced emission characteristic for liver specific imaging with long circulation lifetime. ACS Appl. Mater. Interfaces.

[49] Sakamuru S., Attene-Ramos M.S., Xia M. (2016). Mitochondrial Membrane Potential Assay. Methods Mol. Biol..

[50] Li Y., Zhang R., Wan Q., Hu R., Ma Y., Wang Z., Hou J., Zhang W., Tang B.Z. (2021). Trojan Horse-Like Nano-AIE Aggregates Based on Homologous Targeting Strategy and Their Photodynamic Therapy in Anticancer Application. Adv. Sci..

[51] Wu H., Wang F., Ta N., Zhang T., Gao W. (2021). The Multifaceted Regulation of Mitochondria in Ferroptosis. Life.

