# Supplementary material for: Multiple Light-Activated Photodynamic Therapy of Tetraphenylethylene Derivative with AIE Characteristics for Hepatocellular Carcinoma via Dual-Organelles Targeting
Source: Pharmaceutics. 2022 Feb 21;14(2):459. doi: 10.3390/pharmaceutics14020459 (PMC8877525; doi:10.3390/pharmaceutics14020459)
Supplement: Supplementary file 1 [file pharmaceutics-14-00459-s001.zip › pharmaceutics-1523025-supplementary.pdf]

pharmaceutics

Supplementary Materials: Multiple Light-activated Photodynamic Therapy of Tetraphenylethylene Derivative with AIE Characteristics for Hepatocellular Carcinoma *via* Dual-organelles Targeting

Chuxing Chai, Tao Zhou, Jianfang Zhu, Yong Tang, Jun Xiong, Xiaobo Min, Qi Qin, Min Li, Na Zhao, Chidan Wan

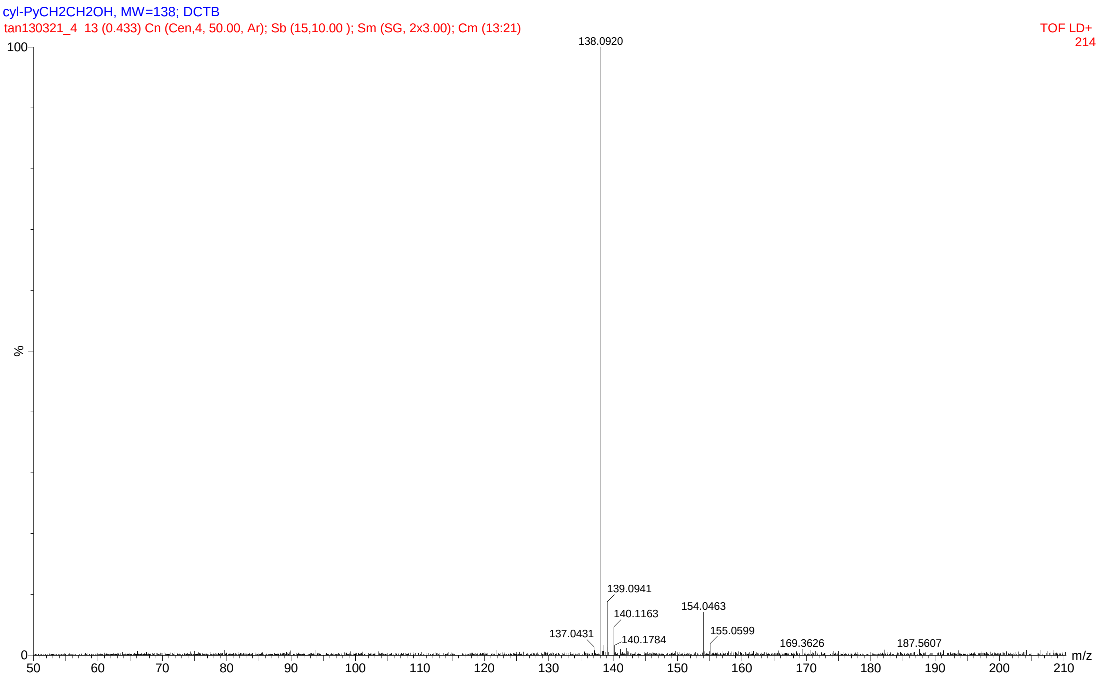

Figure S1. HRMS spectra of molecular **1**.

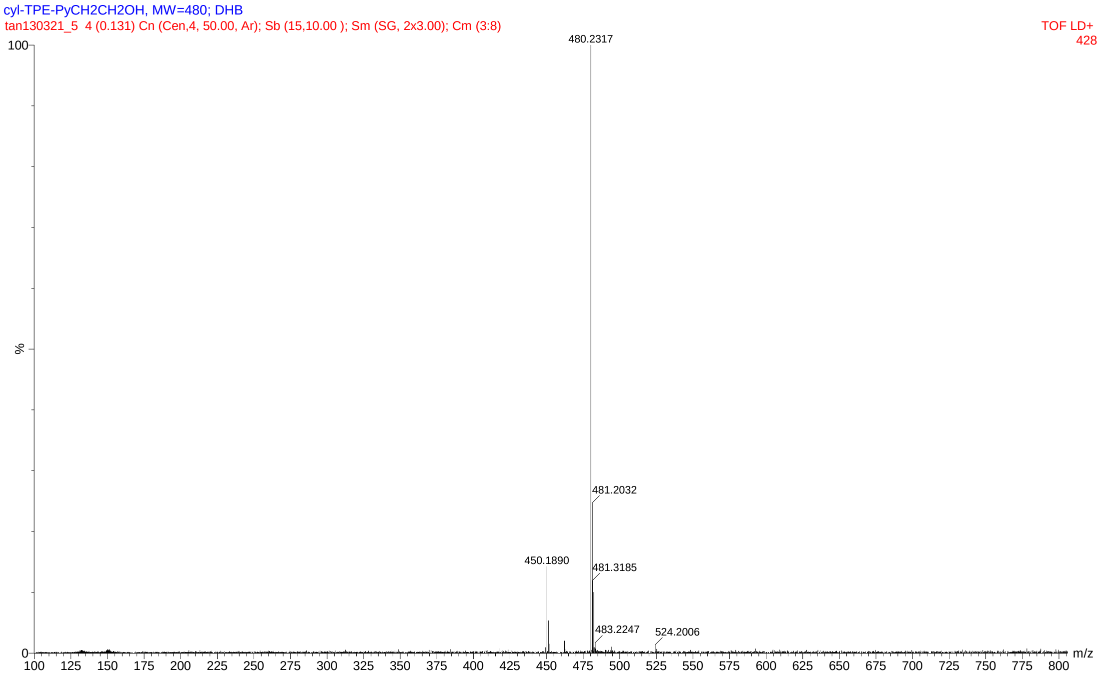

Figure S2. HRMS spectra of TPE-Py-OH.

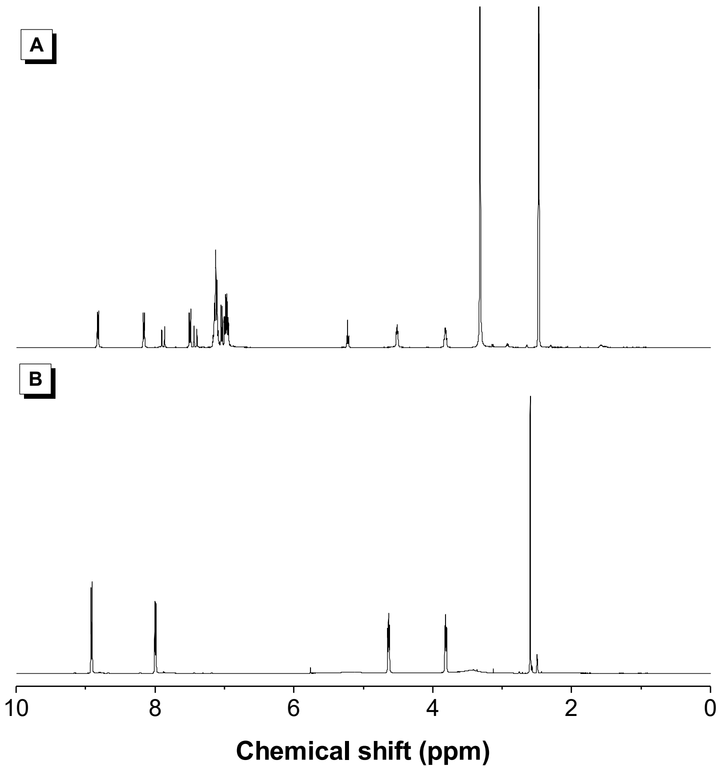

Figure S3. ^{1}H NMR spectra of (**A**) TPE-Py-OH and (**B**) molecular **1** in DMSO-d_6.

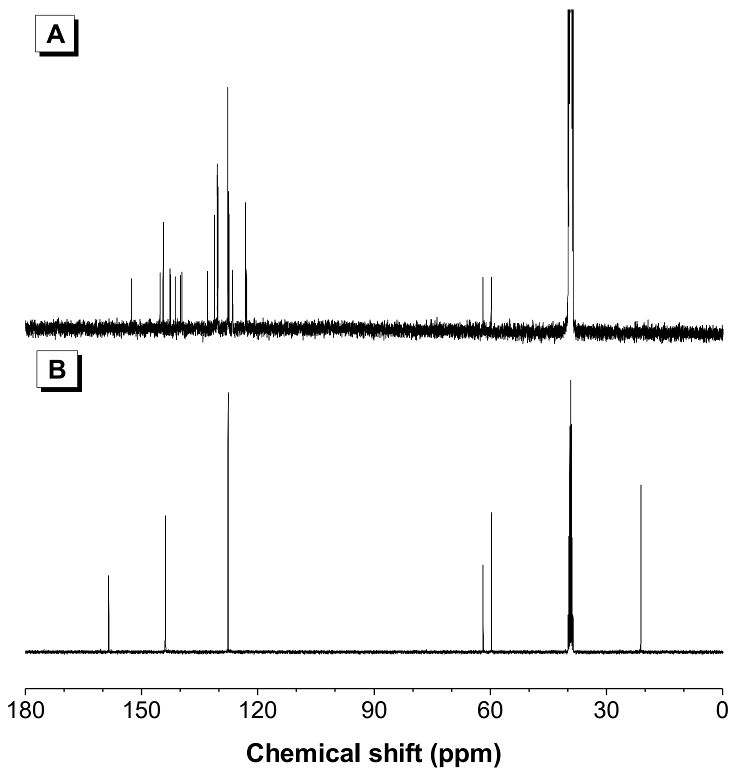

Figure S4. ^{13}C NMR spectra of (**A**) TPE-Py-OH and (**B**) molecular **1** in DMSO-d_6.

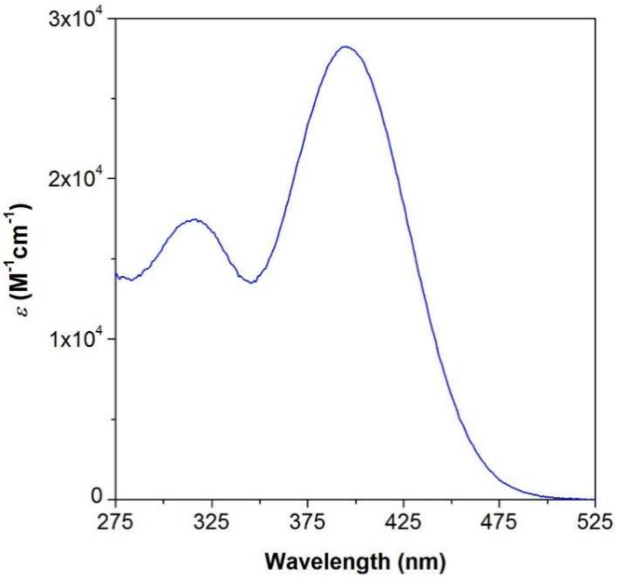

Figure S5. UV spectra of TPE-Py-OH at the concentration of 10 uM in DMSO.

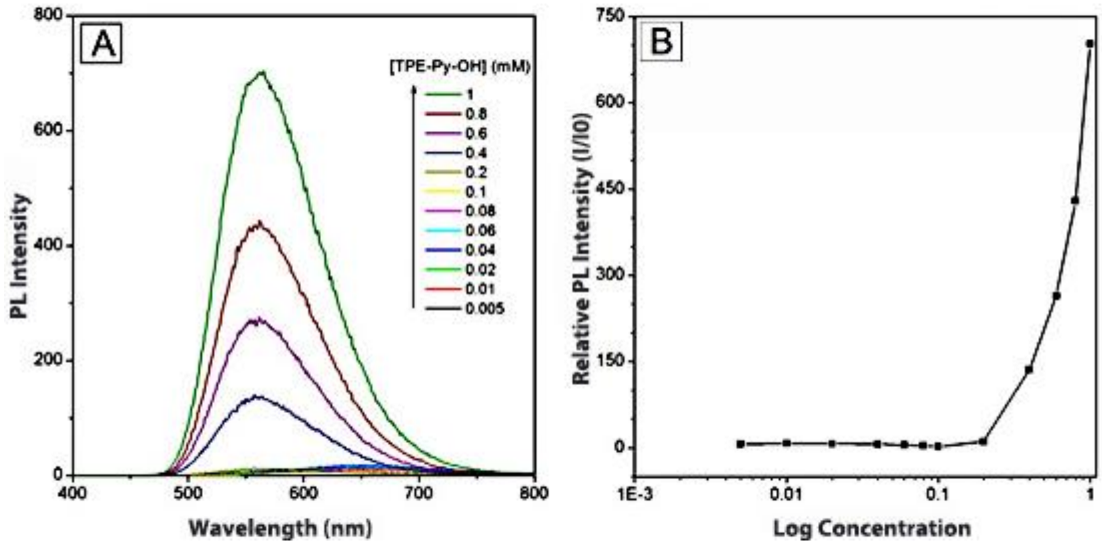

Figure S6. (**A**) PL spectra of TPE-Py-OH in H_2O/DMSO mixtures (*v*/*v* 90%) with different concentrations. Excitation wavelength: 405 nm. (**B**) Plot of PL intensity versus the concentrations of TPE-Py-OH. Inset: photograph of TPE-Py-OH with various concentrations taken under 365 nm UV irradiation.

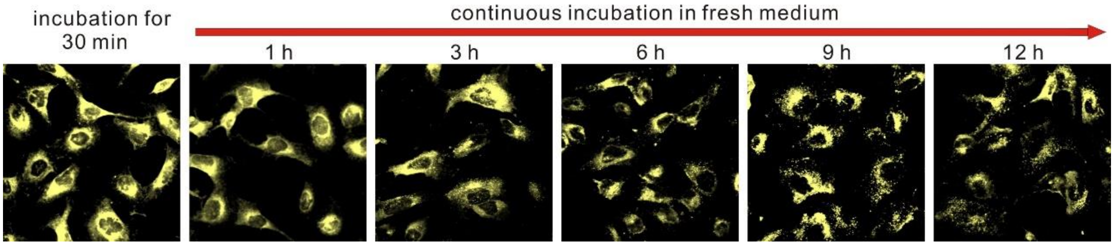

Figure S7. The dynamic monitoring of the distribution of TPE-Py-OH in living cells. The HepG2 cells were incubated with 5 uM TPE-Py-OH for 30 min and then replaced with fresh medium.

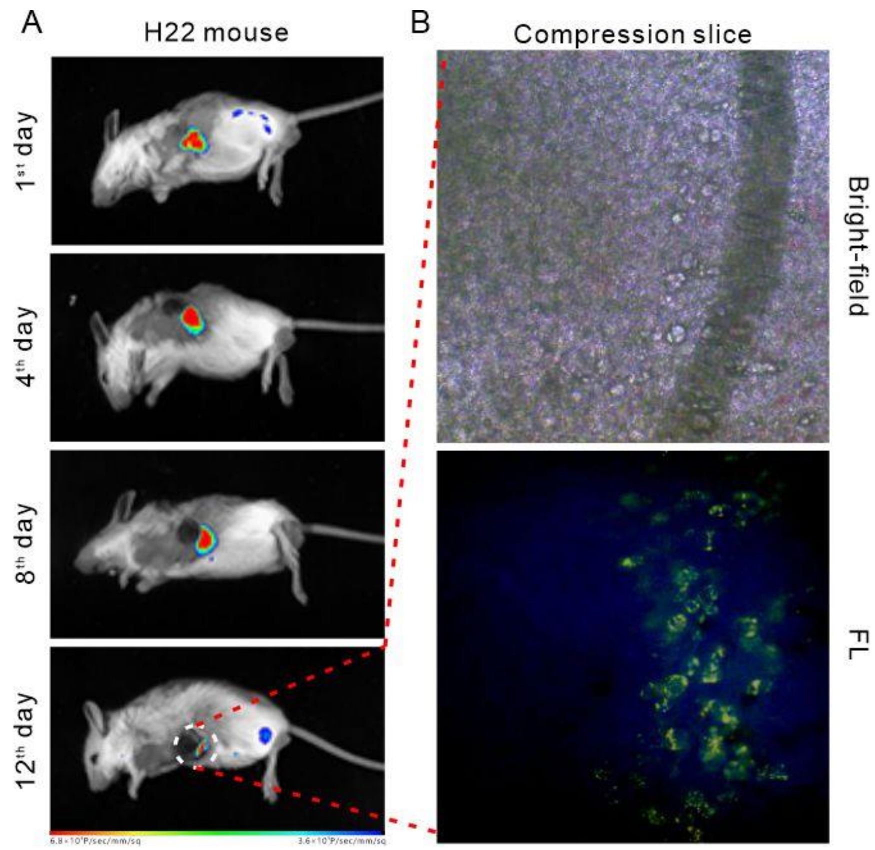

Figure S8. (**A**) The distribution of TPE-Py-OH in vivo after intratumoral injection over times. (**B**) imaging of compression slice of H22 tumor at 12th day after TPE-Py-OH administration.
